# Personalization of Hearing Aid Fitting Based on Adaptive Dynamic Range Optimization

**DOI:** 10.3390/s22166033

**Published:** 2022-08-12

**Authors:** Aoxin Ni, Sara Akbarzadeh, Edward Lobarinas, Nasser Kehtarnavaz

**Affiliations:** 1 Department of Electrical and Computer Engineering, University of Texas at Dallas, Richardson, TX 75080-3021, USA; 2 Callier Center for Communication Disorders, University of Texas at Dallas, Richardson, TX 75080-3021, USA

**Keywords:** personalization of hearing aid fitting, adaptive dynamic range optimization, maximum likelihood inverse reinforcement learning

## Abstract

Adaptive dynamic range optimization (ADRO) is a hearing aid fitting rationale which involves adjusting the gains in a number of frequency bands by using a series of rules. The rules reflect the comparison of the estimated percentile occurrences of the sound levels with the audibility and comfort hearing levels of a person suffering from hearing loss. In the study reported in this paper, a previously developed machine learning method was utilized to personalize the ADRO fitting in order to provide an improved hearing experience as compared to the standard ADRO hearing aid fitting. The personalization was carried out based on the user preference model within the framework of maximum likelihood inverse reinforcement learning. The testing of ten subjects with hearing loss was conducted, which indicated that the personalized ADRO was preferred over the standard ADRO on average by about 10 times. Furthermore, a word recognition experiment was conducted, which showed that the personalized ADRO had no adverse impact on speech understanding as compared to the standard ADRO.

## 1. Introduction

**Figure 1:**
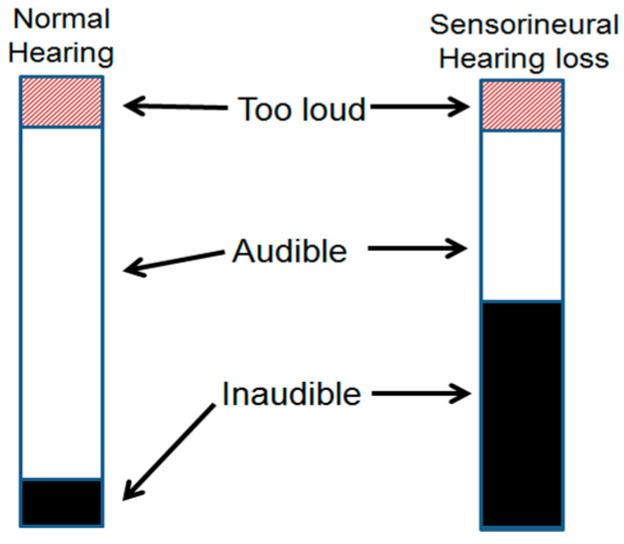
Hearing dynamic range: normal hearing vs. hearing impaired.

Humans have a dynamic range of hearing of about 120 dB sound pressure levels (SPLs). People suffering from hearing loss have a reduced dynamic range of hearing compared to those with normal hearing. Compression is the mechanism by which a reduced dynamic range of hearing is mapped to create the normal dynamic range of hearing during the process of hearing aid fitting [1,2], as demonstrated in Figure 1.

The conventional hearing aid fitting process consists of obtaining a person’s pure-tone hearing thresholds and applying a prescriptive compression or amplification rationale derived from the average values of hearing aid users with similar levels of hearing loss. This process essentially involves applying gain values across a number of frequency bands, depending on the input level of the sound signals. The target gains in the conventional compression or amplification rationales reflect an “average” user and do not take into consideration that different individuals encounter different audio environments and that their hearing preferences in those environments may not be the same.

In order to increase the satisfaction level with hearing aids, some ad hoc self-tuning approaches have been reported in the literature [3,4,5,6]. It is also reported that nearly half of hearing aid users prefer settings that are different than the prescriptive settings [7,8,9]. More recently, machine learning techniques have been utilized to achieve the personalization of hearing aid fitting [10,11,12]. Instead of the conventional approach of using one-size-fits-all gain values, in previous works by our research team, a machine learning approach was developed in order to achieve the personalization of a prescriptive or standard compression or amplification. The motivation for personalizing a prescriptive compression is that hearing preferences differ across individuals and audio environments. In [13,14], a human-in-the-loop deep reinforcement learning (DRL) method was introduced to personalize a widely used prescriptive compression or hearing aid fitting, called DSLv5, via a deep neural network, which was trained based on the preferences expressed by subjects suffering from hearing loss. While the DRL personalization provided promising results, the offline training of its deep neural network posed a limitation for its deployment in real-world audio environments, because its training needed to be performed in an offline manner and could not be performed in an on-the-fly or online manner. In [15], an online personalization method was developed to overcome the offline training shortcomings of the DRL method by using maximum likelihood inverse reinforcement learning (MLIRL). Basically, the novelty of our approach lies in its online training feature. The great majority of machine learning approaches are trained in an offline manner. The online training feature of our approach enables it to be trained via a smartphone app. In other words, the online training feature enables the personalization of ADRO to be carried out in real-world audio environments by a smartphone app in an on-the-fly manner. Another novelty aspect of our personalization approach is that it is applicable to any prescriptive hearing aid fitting rationale and is not limited to any specific prescriptive hearing aid fitting rationale. For example, in [15], our personalization approach was applied to the DSLv5 prescriptive hearing fitting rationale.

In the study reported in this paper, the previously developed online personalization method of MLIRL was applied to a different prescriptive amplification or hearing aid fitting rationale named adaptive dynamic range optimization (ADRO) [16,17,18,19]. ADRO involves selecting a comfort range of the most frequent sound pressive levels based on a number of rules involving the target comfort level of a subject. In ADRO, this comfort range of the most frequent sound pressure levels is obtained with a number of frequency bands based on the estimated SPL percentiles. In this study, the developed personalization of ADRO was then compared to the standard ADRO by carrying out human subject testing involving ten subjects with hearing loss, and the results obtained are reported here.

The rest of the paper is organized as follows. In Section 2, a descriptive overview of the ADRO hearing aid fitting rationale is provided. In Section 3, a descriptive overview of the MLIRL personalization method previously developed by our research team is provided. Section 4 covers our experimental setup for comparing the standard ADRO to the personalized ADRO. The subject testing results and their discussion are then presented in Section 5. Finally, the conclusion appears in Section 6.

## 2. Overview of ADRO

**Figure 2:**
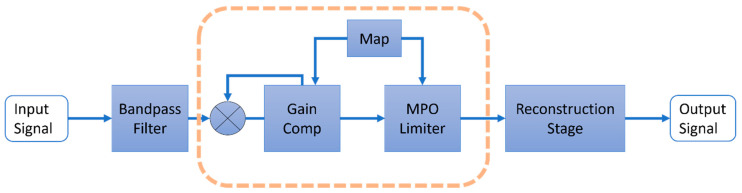
ADRO modules.

Figure 2 provides a block diagram of the ADRO amplification strategy. First, a captured audio signal is decomposed into a number of frequency bands (for example, 5, 10, or 32). The sampling frequency of the input audio signal is often set to 16 kHz. Next, the signal in each frequency band is amplified according to the gain values specified by a gain computation module. This module obtains estimates of the SPL percentiles. For example, the 90th percentile means that 90% of the SPL values are less, and 10% of the SPL values are greater, than a current SPL value over a time period. Three percentiles are normally estimated: the high percentile, mid percentile, and low percentile. These percentiles are compared to the audibility target and comfort target of a user, as defined in the map module, in order to make adjustments to the gain values which are applied to the audio signal in each of the frequency bands. The frequency band components are then combined in order to reconstruct the output audio signal, while staying below the maximum power output (MPO) level.

**Figure 3:**
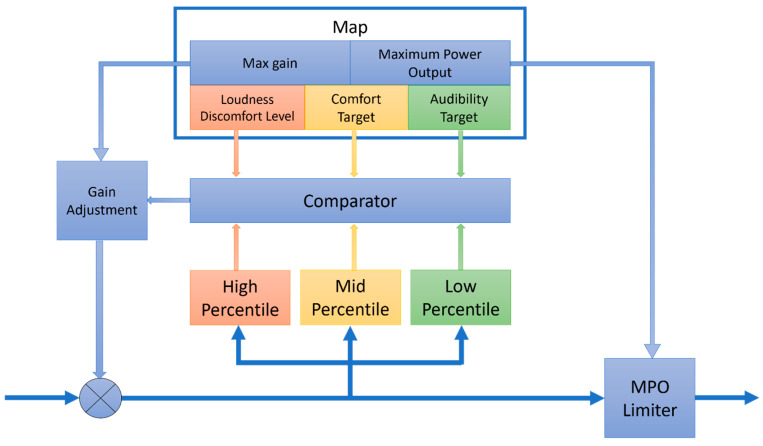
ADRO gain computation module.

The gain computation module is depicted in Figure 3. Three percentiles including the high, mid, and low are estimated from the incoming signals. In [16], these percentiles are specified as the 98th percentile, 70th percentile, and 30th percentile. The estimated percentiles are passed onto a comparator for comparison with the parameters specified in the map module. These parameters consist of the audibility target (AT), comfort target (CT), and loudness discomfort level (LDL) of a person. The audibility level or target denotes the minimum audible threshold level at which a person can hear in each of the frequency bands. Comfort target denotes the level at which audio signals are most effectively heard by a person. This level varies from person to person and from audio environment to audio environment. The loudness discomfort level denotes the sound level perceived to be uncomfortably loud by a person. The MPO parameter is used to limit the output level in each frequency band, so that the signal power does not exceed a maximum value. The max gain (MG) parameter is used to limit the gain value when an update or adjustment is made.

Gain values are adjusted by the comparator according to the following rules: (1) if a high percentile estimate (for example, 90th percentile) exceeds the loudness discomfort level, then the gain value is reduced, for example by 3 dB/s, as specified in [16]; (2) if a mid-percentile estimate (for example, 70th percentile) drops below the comfort target, the gain value is increased, for example by 3 dB/s, as specified in [16], until it becomes equal to the corresponding MG in the map module or the high percentile estimate reaches the LDL in the map module; (3) otherwise, if a low percentile estimate (for example, 30th percentile) goes above the audibility level, then the gain value is reduced by 3 dB/s.

**Figure 4:**
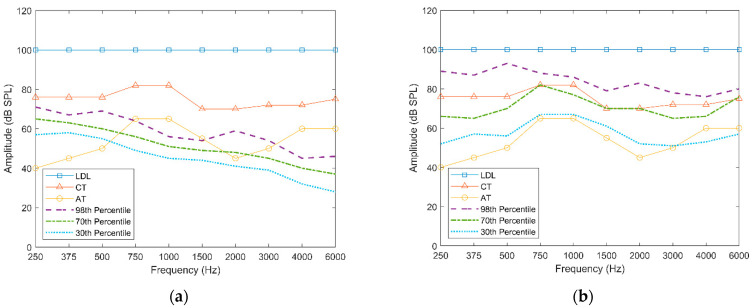
Sample hearing response of a subject: (**a**) SPL percentile distribution before applying ADRO, (**b**) SPL percentile distribution after applying ADRO.

Figure 4 illustrates example gain values before and after applying ADRO. The parameters of the AT and CT used in this example come from one of the subjects reported in the results section who had moderate hearing loss. The region between the LDL and AT denotes the dynamic range of hearing by the subject. In other words, audio signals that fall into this region could be heard by the subject. As shown in Figure 4a, before applying ADRO, most SPL values of the audio signal were below the CT, and the 30th percentile values in the bands of [750 Hz–1500 Hz] and [3000 Hz–6000 Hz] were outside the dynamic range of the subject’s hearing. As shown in Figure 4b, based on the percentile rules applied by ADRO, the audio signal almost fell entirely within the subject’s boundaries of hearing in a more evenly distributed manner. Only a small part of the 30th percentile values which corresponded to high frequencies with relatively low SPLs could not be heard by the subject.

Using the ADRO rules, the gain increase is determined by comparing the 70th percentile and the comfort target. The comfort target has a significant impact on the outcome of ADRO and thus on a subject’s hearing experience. It is worth emphasizing that the comfort target varies from person to person, and it is the parameter personalized in this work. The personalization of the comfort target enables hearing improvement compared to the standard ADRO. In the next section, an overview of the MLIRL personalization applied to the comfort target is described.

## 3. Overview of MLIRL Personalization Method

The details of the developed MLIRL personalization are discussed in our previous work, in [15]. In this section, an overview of this personalization is provided. Mathematical details are not repeated here, and readers are referred to [15,20,21] for such details.

Reinforcement learning (RL) is a machine learning method for pursuing the maximum value of a reward function during its training phase. For many problems, the reward function cannot be easily determined. Inverse reinforcement learning (IRL) enables the obtaining of the reward function based on feedback from a human subject. In the IRL framework, a new action is adopted based on the subject’s response to a personalization state.

The state and action in our case correspond to the comfort targets in a number of frequency bands, denoted by the sets S and A, respectively. These comfort targets, for the standard ADRO, are determined by playing a pure tone in each frequency band and by increasing the SPL of that tone in steps of 5 dB until the subject indicates that it is too loud. Then, the SPL level is reduced by 5 dB, and the subject is asked whether the lower level is more comfortable. If so, that value is assigned as the comfort target for that frequency band. This procedure is repeated for all the frequency bands. Note that the comfort targets obtained above for the standard ADRO are kept fixed and are placed in its map module.

Let (CT_standard_(1), CT_standard_(2), …, CT_standard_(*n*)) represent the standard CTs in the *n* frequency bands. To personalize ADRO, a state/action space is generated by multiplying a scale with the CT in each frequency band as follows:

(1) $${\mathrm{CT}}_{\mathrm{new}}\left(i\right)={\;\mathrm{CT}}_{\mathrm{standard}}\left(i\right)\times \mathrm{scale}\left(i\right)$$

In the training phase, two sets of CTs are selected from the state/action space and are applied to the same audio signal to generate an audio pair. One set of CTs denote a current state s, and the other the action a associated with this state. Then, the audio signal pairs are presented to a subject to select the preferred audio. Based on the subject’s feedback, the reward of applying action a in state s is then updated. The reward function in our case is expressed as ${ℛ}_{W}\left(s,a\right)=W^{T}\varphi \left(s,a\right)$ where $\varphi :S\times A\to {\mathbb{R}}^{n}$ is a known *n*-dimensional state-action function and *W* is an unknown weighting vector of the state-action pairs, which is to be determined. A likelihood function based on a subject’s hearing preferences is defined as follows:

(2) $$L\left(D|W,\;ℋ\right)=\underset{\begin{array}{c} \left(s,\;\;a\right)\in D\; \end{array}}{\prod }\left[\pi_{W}{\left(s,\;a\right)}^{ℋ\left(s,\;a\right)}\right]$$

where D denotes a demonstration consisting of a series of trajectories, with each trajectory denoting a set of state-action pairs $\left\{\left(s_{1},a_{1}\right),\left(s_{2},a_{2}\right),\ldots \right\}$; $\pi_{W}\left(s,\;a\right)$ represents a policy function which reflects the probability of choosing action a in state s; and ℋ denotes a model of a subject’s hearing preferences. The objective of the personalization is to find the optimum W* that maximizes the likelihood of the demonstration, that is:

(3) $$W^{*}=\arg{max}_{W}L\left(D|W,ℋ\right)$$

**Figure 5:**
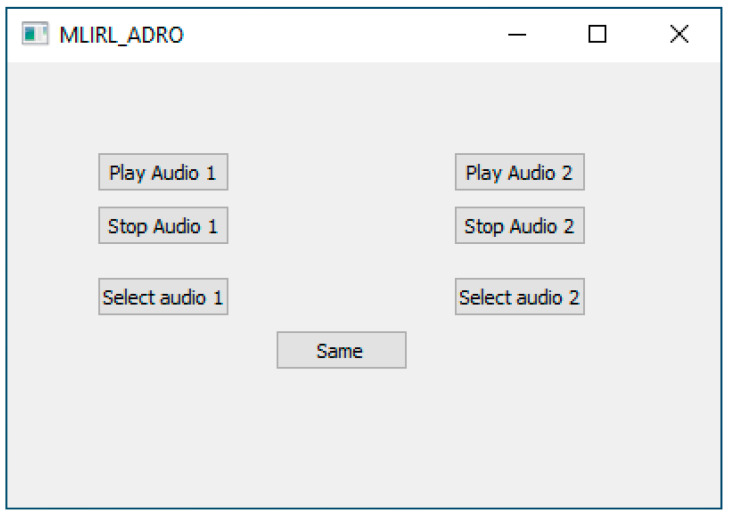
Graphical user interface (GUI) for establishing the preference model of a subject.

The model ℋ is built based on the feedback received from a subject. For this purpose, a speech audio signal is randomly selected from the TSP dataset [22]. This dataset is commonly used in speech processing and consists of over 1400 utterances spoken by 24 speakers (12 females and 12 males). Multi-talker babble noise is added to these utterances at a moderate noise level of 5 dB SNR (signal to noise ratio).The initial state is considered to be CT_standard_. With randomly chosen CTs from the action space A, the first paired comparison in the first trajectory becomes CT_state_ and CT_action_. Hearing preference training is then conducted via the graphical user interface program displayed in Figure 5. The audio signals processed with CT_state_ and CT_action_ are referred to as audio 1 and audio 2 in this interface. The button “same” in the interface is selected when a subject indicates no preference between the audio signals in a pair.

**Figure 6:**
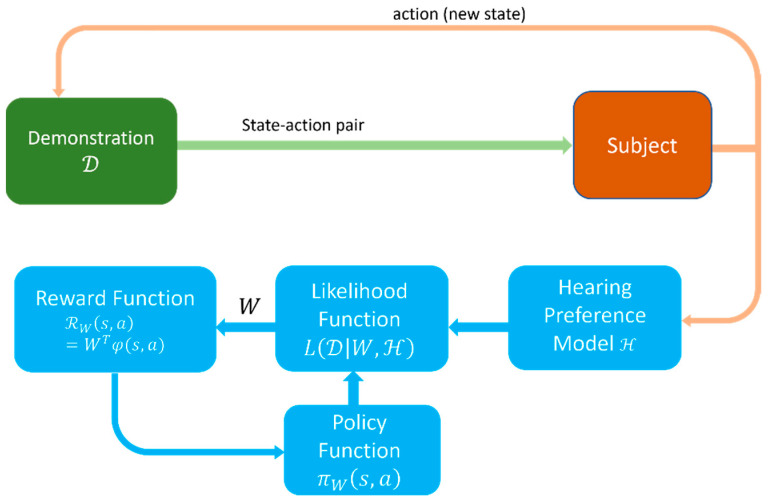
Components of the MLIRL personalization method.

If a subject selects CT_action_ as the preferred audio signal, then the feedback for choosing CT_action_ under CT_state_ is considered to be positive. Otherwise, the feedback is considered to be negative. The feedback becomes neutral when no difference between audio 1 and audio 2 is indicated by the subject during the online training process. Based on all such feedback received from a subject over the demonstration, a preference model ℋ of that subject is established. Then, this preference model is set in the likelihood function and the derivative of the likelihood function is set to zero in order to find the maximum or optimum reward function W*. The personalized comfort targets are assigned to be the ones corresponding to the optimum reward W*. The interested reader is referred to [15,20] for the mathematical details of the training process. The components of the MLIRL personalization method are illustrated in Figure 6.

## 4. Experimental Setup

This section covers our experimental setup for the subject testing results reported in Section 5. Ten subjects with mild to moderately severe hearing loss were recruited to participate in this study under the approved human subject Institutional Review Board protocol (IRB 20-13) at the University of Texas in Dallas. Eligibility for the participating subjects included: (i) symmetric mild to moderately severe hearing loss, (ii) being able to speak and understand English, and (iii) being an adult in the age range of 21–80 years old capable of providing an informed consent.

All the experimentations were performed in a sound booth in three sessions. The most critical amplification was performed for voice signals which reside in the 4 kHz range. Nearly all prescriptive compressions operate in this range. For example, in the prescriptive ADRO, the audiogram and the nominal comfort targets are set for the following ten frequency bands: [125 Hz–250 Hz], [250 Hz–500 Hz], [500 Hz–750 Hz], [750 Hz–1000 Hz], [1000 Hz–1500 Hz], [1500 Hz–2000 Hz], [2000 Hz–3000 Hz], [3000 Hz–4000 Hz], [4000 Hz–6000 Hz], and [6000 Hz–higher]. In the first session, the audiogram and the nominal comfort targets of a subject for the above ten frequency bands were measured. Then, these measured values were used to initialize the ADRO hearing aid fitting or to set the standard ADRO amplification. To increase the efficiency of the the training process of MLIRL, the comfort targets were mapped into the following five frequency bands: [0 Hz–500 Hz], [500 Hz–1000 Hz], [1000 Hz–2000 Hz], [2000 Hz–4000 Hz], [4000 Hz–higher].

**Figure 7:**
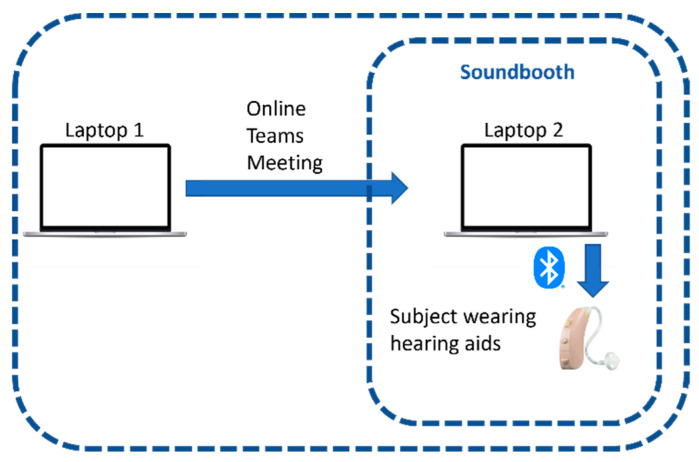
Experimental sound booth setup.

The second session involved the online training session of the MLIRL personalization. As depicted in Figure 7, each subject sat in the sound booth wearing a pair of commercial hearing aids with Bluetooth connectivity capability. A Bluetooth wireless connection was established between the hearing aids and a laptop in the sound booth (laptop 2 in Figure 7). To communicate and guide the subject, another laptop (laptop 1 in Figure 7) was used by the experimenter outside of the sound booth connected to laptop 1 via the Teams online meeting the subject was visually monitored through a window. Speech signals of the TSP dataset were used for the training session. The TSP dataset contains over 1400 utterances spoken by 24 speakers, both females and males. The content of each utterance is a short sentence with a duration of about 2.5 s. A moderate level of multi-talker babble noise (SNR of 5 dB) was added to the speech signals. The sampling frequency was set to 16 kHz to ensure that the speech content with the highest frequency of about 4 kHz was reconstructed with no distortion. Audio pairs were played to a subject and the subject was asked to indicate his or her hearing preference by selecting audio 1 or audio 2 (see Figure 5). In case there was no preference between an audio pair, the GUI provided the option of choosing “same”. The only difference between the audio pairs was the use of different comfort targets (CTs). The outputs for each audio pair were normalized to ensure that the only difference between them was due to their different comfort targets. To keep the training time below 2 h, and thus to avoid human fatigue in training the personalization algorithm, the training session was limited to seven trajectories of 31 paired comparisons.

After the completion of the training session, a testing session was conducted. The testing involved playing thirty audio pairs that were randomly selected from the TSP dataset and with the added babble noise. An audio pair played during the testing session consisted of the standard ADRO and the personalized ADRO. The order of presenting an audio pair was randomized, i.e., sometimes the standard ADRO was played first and sometimes the personalized ADRO was played first. The subject was then asked to select the preferred audio.

## 5. Results and Discussion

This section discusses the outcomes of the subject testing that was conducted. These results are shown in Table 1. In this table, the audiograms of the participating subjects, together with their comfort targets for both the standard ADRO and the personalized ADRO across the ten frequency bands considered, are listed. This table was generated after the online training session. Here, it is worth emphasizing that, in the personalized ADRO, every two consecutive frequency bands corresponded to one training frequency band.

**Figure 8:**
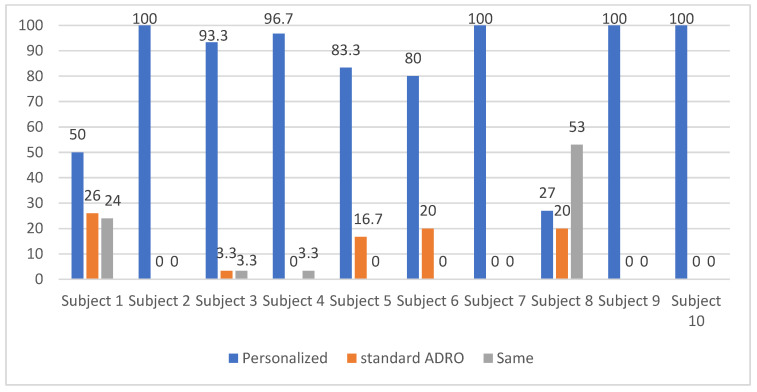
Comparison of the preference percentages between the standard ADRO and the personalized ADRO.

The outcomes of the testing session are provided in Figure 8. As shown in Figure 8, the total number of utterances preferred by the personalized ADRO across all the ten subjects was 830, and the total number of utterances preferred by the standard ADRO was 86. In other words, the personalized ADRO was preferred over the standard ADRO by about 10 times. A one-way ANOVA statistical test with a *p*-value less than 0.01 was computed. In this test, the so-called F statistic value with 1 and 18 degrees of freedom became 74.3, indicating that the hearing preference for the personalized ADRO was statistically significant. The interested reader is referred to [23] for the mathematical details of this statistical test.

**Figure 9:**
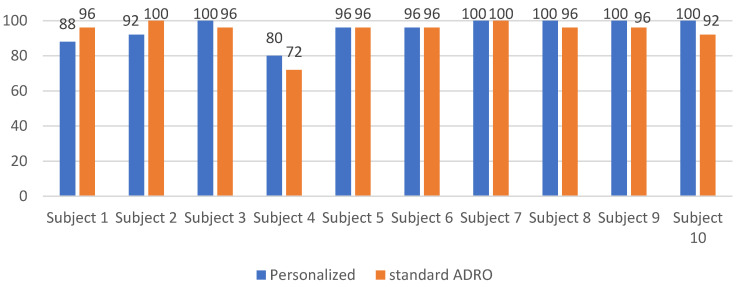
Word recognition scores of the standard and personalized ADRO as percentages in quiet conditions.

**Figure 10:**
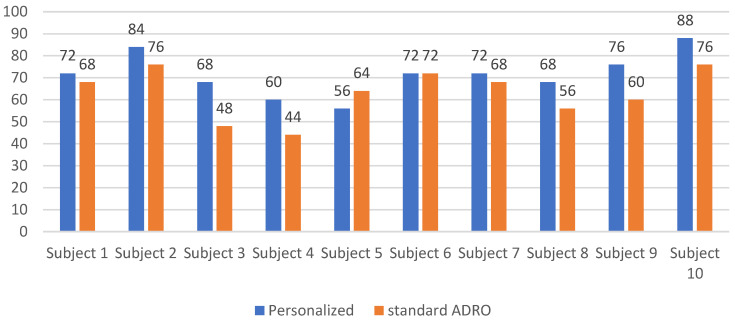
Word recognition scores of the standard and personalized ADRO as percentages in noisy conditions at 5 dB SNR.

Another experiment was conducted to examine whether any negative impact on the word recognition or speech understanding was caused by the personalized ADRO settings as compared to the standard ADRO settings. Two 50-word lists were selected from the Northwestern University Auditory Test No. 6 (NU-6) dataset [24]. One list was played with no noise (quiet) and the other was played by adding babble background noise at 5 dB SNR. For each word list, half of the words were played using the standard ADRO, while the other half were played using the personalized ADRO. The order in which the words were played to the subjects was randomized. The subjects were asked to repeat each word back to the examiner after it was presented to them just once. When the correct word was stated, the word recognition score was increased by one. The word recognition scores obtained are shown in Figure 9 and Figure 10, respectively, in the quiet and noisy conditions for the ten participating subjects in the study. As can be seen from these figures, in the quiet condition, the subjects could recognize almost all the words, with practically no difference between the standard ADRO and the personalized ADRO, while in the noisy condition, there was a slight increase in the average word recognition score when using the personalized ADRO. The slight increase in the average word recognition score was due to the fact the personalized ADRO provided a better amplification of the audio signals over the standard ADRO, leading to the subject recognizing more words correctly. The point of this experiment was solely to ensure that the personalized ADRO would not adversely impact the word recognition score compared to the standard ADRO.

## 6. Conclusions

This paper presented the personalization of a hearing aid fitting rationale based on adaptive dynamic range optimization (ADRO). A previously developed personalization method based on maximum likelihood inverse reinforcement learning (MLIRL) was utilized to personalize the ADRO. This was achieved by personalizing the comfort target levels across five frequency bands via an online training session. To demonstrate the engineering feasibility or proof-of-concept of this personalization method, subject testing experiments were conducted on ten participating subjects. The results obtained indicated that the personalized ADRO was preferred over the standard ADRO on average by about 10 times. Furthermore, it was shown that the personalized ADRO had no adverse impact on speech understanding as compared to the standard ADRO. The online training feature of the developed personalization method allows it to be easily deployed in the field. In our future work, we plan to carry out this personalization online training in the field or in real-world audio environments by developing a smartphone app.

## Figures and Tables

**Table 1:** Personalized ADRO comfort targets after online training by MLIRL.

Subject	Audiogram (dB)	Comfort Targets (CTs) of Standard ADRO (dB)	Comfort Targets (CTs) of Personalized ADRO (dB)
1	(20, 20, 15, 15, 15, 15, 15, 15, 25, 40)	(70, 70, 70, 70, 72, 75, 77, 80, 75, 70)	(35, 35, 35, 35, 55, 75, 57, 40, 37, 35)
2	(5, 10, 10, 15, 15, 15, 15, 20, 20, 30)	(70, 70, 75, 80, 80, 80, 80, 80, 77, 75)	(35, 35, 37, 40, 60, 80, 80, 80, 77, 75)
3	(20, 25, 15, 25, 25, 25, 20, 20, 30, 35)	(75,75, 75, 75, 72, 70, 75, 80, 72, 65)	(75, 75, 75, 75, 55, 35, 57, 80, 72, 65)
4	(45, 40, 40, 40, 40, 45, 40, 35, 45, 50)	(78, 78, 80, 82, 81, 80, 80, 80, 77, 75)	(39, 39, 40, 41, 40, 40, 60, 80, 58, 37)
5	(40, 45, 50, 65, 65, 55, 45, 50, 60, 60)	(76, 76, 79, 82, 76, 70, 71, 72, 73, 75)	(38, 38, 60, 82, 58, 35, 53, 72, 54, 37)
6	(20, 20, 20, 20, 25, 25, 30, 40, 50, 45)	(78, 78, 80, 82, 79, 77, 78, 80, 80, 80)	(39, 39, 59, 80, 59, 38, 59, 80, 80, 80)
7	(15, 15, 25, 25, 30, 30, 30, 25, 40, 45)	(76, 76, 79, 82, 82, 82, 79, 77, 71, 65)	(38, 38, 39, 41, 41, 41, 59, 77, 71, 65)
8	(40, 30, 20, 20, 10, 10, 20, 50, 55, 40)	(82, 82, 77, 72, 71, 70, 72, 75, 70, 65)	(41, 41, 38, 36, 35, 35, 36, 37, 34, 32)
9	(15, 15, 10, 20, 20, 20, 20, 35, 50, 40)	(80, 80, 81, 82, 78, 75, 80, 85, 82, 80)	(40, 40, 40, 41, 39, 37, 39, 42, 61, 80)
10	(10, 15, 10, 35, 45, 45, 30, 25, 15, 20)	(56, 56, 61, 67, 67, 67, 66, 65, 57, 50)	(28, 28, 30, 33, 33, 33, 32, 32, 41, 50)

## Data Availability

Not applicable.
